# Supplementary material for: Chitinase-3-like Protein 1 Is Associated with Poor Virologic Control and Immune Activation in Children Living with HIV
Source: Viruses. 2022 Nov 23;14(12):2602. doi: 10.3390/v14122602 (PMC9786985; doi:10.3390/v14122602)
Supplement: Supplementary file 1 [file viruses-14-02602-s001.zip › viruses-1991030-supplementary.pdf]

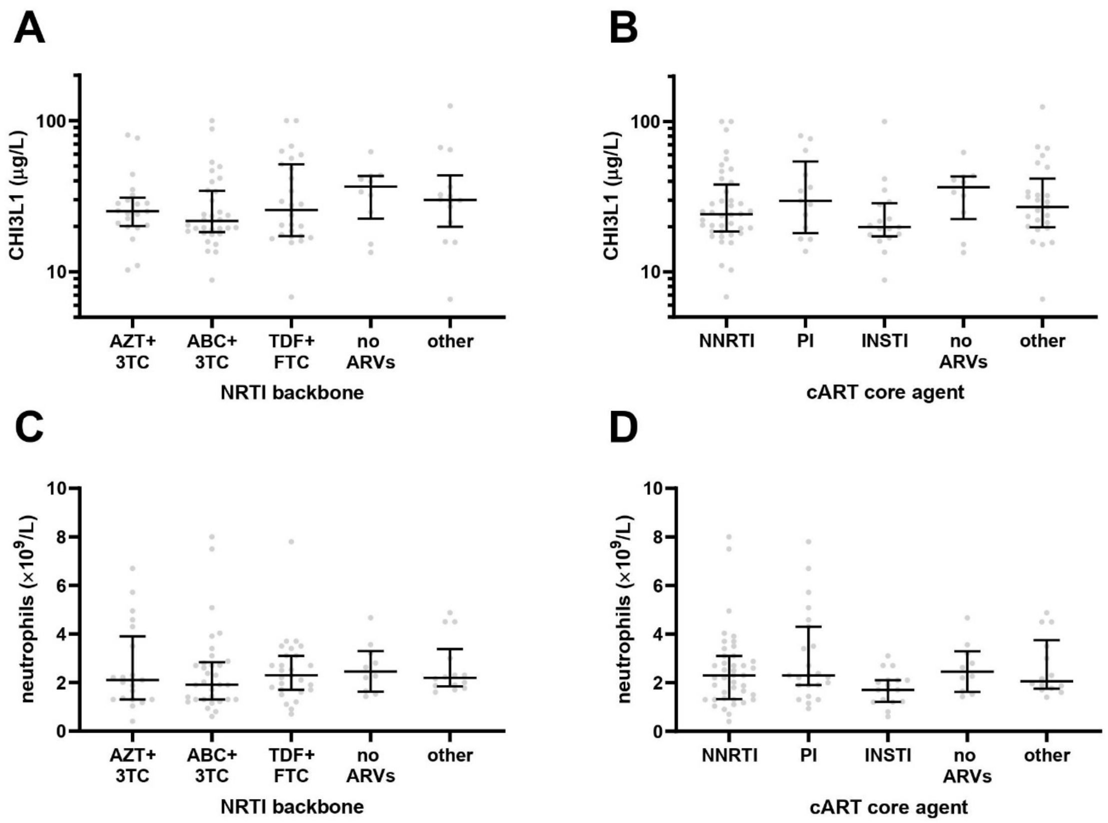

Figure S1. cART regimen is not associated with differences in CHI3L1 and neutrophil count. A. CHI3L1 levels (p=0.45) and **B.** neutrophil counts (p=0.78) did not differ significantly between NRTI backbone combinations. AZT, zidovudine; ABC, abacavir; 3TC, lamivudine; TDF, tenofovir; FTC, emtricitabine. Other NRTI components of the cART regimen included: ABC+TDF+FTC (n=1); ABC+TDF+3TC (n=1); ABC+AZT (n=2); ABC (n=2); 3TC (n=2); AZT (n=1); no NRTI in cART regimen (n=2); and unknown (n=5). **C.** CHI3L1 levels (p=0.27) and **D.** neutrophil counts (p=0.13) did not differ significantly between cART regimen, according to core agent. NNRTI, non-nucleoside reverse transcriptase inhibitor, PI, protease inhibitor (includes ritonavir-boosted PIs); INSTI, integrase strand-transfer inhibitor.
